# Serum fetuin-A, tumor necrosis factor alpha and C-reactive protein concentrations in patients with hereditary angioedema with C1-inhibitor deficiency

**DOI:** 10.1186/s13023-019-0995-7

**Published:** 2019-03-18

**Authors:** Bernadett Márkus, Nóra Veszeli, György Temesszentandrási, Henriette Farkas, László Kalabay

**Affiliations:** 1 Department of Family Medicine, Semmelweis University, Budapest POB 2, Kútvölgyi str. 4, Budapest, H-1125 Hungary; 2 Hungarian Angioedema Reference Center, 3rd Department of Internal Medicine, Semmelweis University, Budapest, Hungary; 3 Semmelweis University, POB 2, Budapest, H-1428 Hungary

**Keywords:** Fetuin-A, Tumor necrosis factor alpha, C-reactive protein, Hereditary angioedema, C1 inhibitor deficiency

## Abstract

**Background and aims:**

Hereditary angioedema with C1-inhibitor deficiency (C1-INH-HAE) is characterized by localized, non-pitting, and transient swelling of submucosal or subcutaneous region. Human fetuin-A is a multifunctional glycoprotein that belongs to the proteinase inhibitor cystatin superfamily and has structural similarities to the high molecular weight kininogen. Fetuin-A is also known a negative acute phase reactant with anti-inflammatory characteristics. In this study we aimed to determine serum fetuin-A, C-reactive protein (CRP) and tumor necrosis factor alpha (TNFα) concentrations in patients with C1-INH-HAE during symptom-free period and during attacks and compare them to those of healthy controls. Further we analyzed possible relationship among these parameters as well as D-dimer levels which was known as marker of HAE attacks.

**Patients and methods:**

Serum samples of 25 C1-INH-HAE patients (8 men, 17 women, age: 33.1 ± 6.9 years, mean ± SD) were compared to 25 healthy controls (15 men, 10 women, age: 32.5 ± 7.8 years). Serum fetuin-A and TNFα concentrations were determined by ELISA, CRP and D-dimer by turbidimetry.

**Results:**

Compared to healthy controls patients with C1-INH-HAE in the symptom-free period had significantly decreased serum fetuin-A 258 μg/ml (224–285) vs. 293 μg/ml (263–329), (median (25–75% percentiles, *p* = 0.035) and TNFα 2.53 ng/ml (1.70–2.83) vs. 3.47 ng/ml (2.92–4.18, *p* = 0.0008) concentrations. During HAE attacks fetuin-A levels increased from 258 (224–285) μg/ml to 287 (261–317) μg/ml (*p* = 0.021). TNFα and CRP levels did not change significantly. We found no significant correlation among fetuin-A CRP, TNFα and D-dimer levels in any of these three groups.

**Conclusions:**

Patients with C1-INH-HAE have decreased serum fetuin-A concentrations during the symptom-free period. Given the anti-inflammatory properties of fetuin-A, the increase of its levels may contribute to the counter-regulation of edema formation during C1-INH-HAE attacks.

## Introduction

Hereditary angioedema with C1-inhibitor (C1-INH) deficiency (C1-INH-HAE) is a rare autosomal dominant disorder (estimated prevalence: 1:150,000 to 1:10,000) [1] characterized by the decreased of C1 inhibitor (C1-INH) activity. In 80% of the cases the C1-INH molecule has low antigenic levels (C1-INH-HAE Type I), in 20% C1-INH is present and can have high antigen levels but with low function (C1-INH-HAE Type II). C1-INH regulates the complement, contact, coagulation, and fibrinolytic plasma enzyme cascades. The deficiency of C1-INH leads to the uncontrolled, spontaneous activation of these plasma enzyme systems. Contact-kinin system activation results in the release of the vasoactive mediator bradykinin from high molecular weight kininogen (HMWK), which causes vasodilation, increased vascular permeability, and plasma leakage into the extracellular space, leading to edema formation [2–4]. The HAE attacks may involve the extremities, the face, the trunk genitals, and submucosal tissues in the gastrointestinal tract and upper airways. In the gastrointestinal tract, angioedema may mimic an abdominal catastrophe, whereas in the upper airways, it may cause obstruction leading to suffocation [5]. Occurrence of HAE attacks is unpredictable, but some trigger factors, including infection, mechanical trauma, mental stress, hormonal changes, drugs (estrogens and angiotensin converting enzyme inhibitors) can be explored in a proportion of patients [6, 7].

Recently, some evidences tend to confirm a relationship between HAE and atherosclerosis, as well. In this respect, Demirtürk et al. observed decreased coronary blood flow reserve pointing to increased risk of atherosclerosis [8]. Moreover, in their latest paper Firinu et al. observed impaired finger plethysmography values and asymmetric dimethylarginine levels strongly suggesting endothelial dysfunction in this disease [9].

Bradykinin-mediated angioedema should be distinguished from histaminergic angioedema. The latter is characterized by the type I immunoreaction, rapid (24 h) symptom development, frequent association with itching urticaria, and responsiveness to antihistamines, corticosteroids or epinephrine. Bradykinin-mediated angioedema has a more protracted symptom development (typically 3–5 days), does not present with pruritus but can be painful, and does not react to the drugs mentioned above. The acute HAE attacks are terminated by C1-INH concentrate and tranexamic acid and danazol are established for prophylaxis.

Human fetuin-A (formerly called α2HS-glycoprotein) is a multifunctional glycoprotein which is secreted almost exclusively by the liver parenchymal cells in adulthood [10]. Early studies have shown that fetuin-A acts as a negative acute phase protein [11], decreases the phytohemagglutinin-induced lymphoblastic transformation [12], increases opsonization and phagocytosis [13, 14] and regulates superoxide release of neutrophil granulocytes [15].

In addition fetuin-A is a mineral chaperone [16], attaches to hydroxyapatite crystals and inhibits calcification both in vitro and in vivo [15, 17]. It accumulates is bone being the most abundant non-collagenous protein in bone and dentin [10, 18]. The role of fetuin-A has also been established in the development of obesity [19, 20], insulin resistance [21], metabolic syndrome [20, 22], adipocyte dysfunction [23], fatty liver [21], and type 2 diabetes [24, 25].

Probably due to impaired inhibition of vascular calcification low serum fetuin-A concentration has been associated with increased cardiovascular risk in patients without diabetes, as well [26, 27].

Fetuin-A is a member of the cystatin superfamily [28, 29]. Cystatins are proteinase inhibitors. This superfamily has members with similar tandem repeats, one in cystatin C, two in fetuin-A and fetuin B, and three in the kininogens [29]. In fetuin-A the proline-rich carboxyl-terminal region of the A-chain displays sequence similarity to collagens and the collagen-like domains of complement component C1q [30].

The serum fetuin-A has not been investigated in C1-INH-HAE; however, these structural similarities of fetuin-A with C1q and HMWK, which have important role in the pathomechanism of C1-INH-HAE and as fetuin-A is a negative acute phase protein may influence the development of HAE attacks. Therefore, we aimed to determine serum concentrations of fetuin-A, and other inflammatory markers such as C-reactive protein (CRP) and tumor necrosis factor-α (TNFα) in patients with C1-INH-HAE during both symptom-free period and attacks and compare them to those of healthy controls.

## Patients and methods

### Patients and controls

Twenty-five C1-INH-HAE patients (8 men, 17 women, age: 33.1 ± 6.9 years, mean ± SD), 20 with type I and 5 patients with type II of C1-INH-HAE, were enrolled to our study. The diagnosis of C1-INH-HAE was established by pedigree-analysis, as well as by the evaluation of the clinical manifestations and complement parameters (low antigenic and functional levels of C1-INH, low level of C4 and normal level of C1q). Ten patients received long-term prophylaxis, 9 of them were on long-term danazol, and one of them was on tranexamic acid. The remaining 15 patients did not receive long-term prophylaxis. For acute treatment of HAE attacks patients received human plasma-derived C1-INH concentrate (Berinert®, CSL Behring, Marburg, Germany) when it was necessary. The location of the HAE attack, the onset of edematous symptoms as well as the time from onset to acute treatment were recorded in the Hungarian HAE Registry. Twelve HAE attacks occurred submucosally (7 in abdominal viscera, 3 in the upper airways, 2 in other localization), 12 subcutaneously, and 1 in mixed locations.

The control group consisted of 25 healthy volunteers (10 men, 15 women, age: 32.5 ± 7.8 years), referred for routine medical evaluation. The healthy controls did not have any known disease, nor they received medicinal products at the time of blood sampling. C1-INH deficiency was excluded by complement testing. The C1-INH-HAE patients and the controls were not statistically different as regards age and gender distribution.

### Blood sampling

Peripheral blood samples were obtained from patients with C1-INH-HAE both during symptom-free periods and during attacks (before acute treatment). “Symptom-free” samples were obtained during the annual control visits in the Hungarian Angioedema Center. “During attack” samples were obtained prior to acute treatment, within 6 h after the onset of the edematous symptoms. None of the patients had any clinical manifestations suggestive of an acute infection during the HAE attack. Peripheral blood samples were drawn also from healthy subjects. According to standard procedures native serum (after clotting completed), EDTA- and citrate-anticoagulated plasma (immediately after blood taking) were separated by centrifugation at 3500 rpm for 10 min. Thereafter, the obtained serum, EDTA and citrate plasma samples were then stored below − 70 °C until processing.

### Methods

All analyzed parameters were determined using the same unthawed aliquot from each subject, and each assay was performed on aliquots thawed for the same length of time. Plasma fetuin-A and TNFα concentrations were determined by sandwich-type ELISA (BioVendor, Czech Republic, and Thermofisher Scientific Inc., Waltham, USA, respectively), according to the manufacturers’ instructions. Levels of CRP were determined in EDTA-plasma samples using a chemistry analyzer (Beckman Coulter Inc., California, USA).

The determination of D-dimer concentration was performed in citrated plasma by latex agglutination immunoturbidimetry on a COAG XL coagulometer (Diagon Ltd., Budapest, Hungary) using the Dia-D-DIMER test (Diagon Ltd., Budapest, Hungary).

### Statistical analysis

The statistical analysis was performed by the SPSS 23 version (SPSS, Chicago, IL, USA). We used nonparametric tests throughout the analysis. All the statistical analyses were two-tailed, and *p* < 0.05 was considered to represent a significant difference, or correlation.

## Results

During the symptom-free period C1-INH-HAE patients had significantly lower fetuin-A and TNFα levels compared to healthy controls. CRP levels did not show marked differences compared these two groups (Table 1). In “during attack” samples of C1-INH-HAE patients fetuin-A levels were significantly higher compared to the symptom-free period of the same patients. In contrast, CRP and TNFα levels were comparable in samples obtained from symptom-free and from during attack periods of the same patient. D dimer levels significantly rose in patients during attacks than in the symptom-free period of the same patients and were also higher compared to the healthy control group.

**Table 1:** Serum fetuin-A, CRP and TNFα concentrations in patients with C1-INH-HAE and healthy controls

	Symptom-free phase	Attack	Healthy controls	p1	p2	p3
Fetuin-A, μg/ml	258	287	293	0.035	0.021	0.945
	(224–285)	(261–317)	(263–329)			
CRP, mg/l	1.25	3.57	1.95	0.303	0.247	0.528
	(0.77–4.75)	(0.93–4.73)	(1.16–3.91)			
TNFα, ng/ml	2.53	2.73	3.47	0.0008	0.207	0.166
	(1.70–2.83)	(1.80–3.71)	(2.92–4.18)			
D-dimer, μg/ml	0.52	2.44	0.42	0.405	0.023	0.006
	(0.22–1.26)	(0.67–5.36)	(0.36–0.85)			

Values in median (25–75) percentiles

p1: symptom-free phase vs. healthy controls, Mann-Whitney test

p2: symptom-free phase vs. attack, Wilcoxon test

p3: attack vs. healthy controls, Mann-Whitney test

We divided our patients according to the localization of HAE attack (Table 2). Dividing the measured parameters regarding attack location we found elevated fetuin-A levels only during subcutaneous attacks compared to symptom-free period: 295 (260–325) μg/ml vs. 254 (200–273) μg/ml, *p* = 0.033; median (25–75 percentile) (*n* = 12), whereas during submucosal attacks (abdominal plus upper airways localization) fetuin-A levels the difference between “during HAE attacks” and “symptom-free” samples was not statistically significant: 286 (262–320) ug/ml vs. 265 (241–297) ug/ml, *n* = 12, *p* = 0.308.

**Table 2:** Comparison of serum fetuin-A, CRP and TNFα concentrations in patients with C1-INH-HAE with different attack localization

	Symptom-free phase	Attack	*p* ^#^
Subcutaneous attack (*n* = 12)			
Fetuin-A, mg/l	254 (200–273)	295 (260–325)	0.033
CRP, mg/l	1.19 (0.99–6.16)	3.61 (0.79–4.44)	0.722
TNFα, ng/ml	2.19 (1.70–2.83)	2.73 (1.75–3.71)	0.237
D-dimer, μg/ml	0.52 (0.22–0.74)	1.15 (0.41–4.47)	0.110
Submucosal attack (*n* = 12)			
Fetuin-A, mg/l	265 (241–297)	286 (262–320)	0.308
CRP, mg/l	1.84 (0.71–4.41)	3.64 (1.34–5.66)	0.272
TNFα, ng/ml	2.58 (1.11–2.88)	3.17 (1.80–4.75)	0.310
D-dimer, μg/ml	0.72 (0.17–2.19)	2.44 (1.07–16.52)	0.116

Values in median (25–75) percentiles

^#^Wilcoxon-test

We did not observe significant differences in fetuin-A, CRP or TNFα levels between subcutaneous and submucosal groups. Comparison of serum fetuin-A, CRP and TNFα concentrations during HAE attacks with healthy controls showed no significant differences.

We found no significant correlations among fetuin-A, CRP,TNFα and D-dimer levels in any of these three groups (data not shown).

Fetuin-A, CRP and TNFα levels of patients on long-term prophylaxis did not differ from those of not receiving it.

## Discussion

To our best knowledge serum fetuin-A has not been investigated in patients with C1-INH-HAE. Compared to healthy controls we observed significantly decreased serum fetuin-A concentration levels in C1-INH-HAE patients. This phenomenon cannot be explained by the negative acute phase character of the molecule [11, 31] since CRP and TNFα did increase accordingly. Our patients had no documented infection at the time of HAE attacks. Since fetuin-A levels did not correlate neither with the positive acute phase protein CRP, nor with D-dimer or TNFα concentrations in any groups one might suppose that alteration of fetuin-A level is independent of the acute phase reaction.

Furthermore, TNFα levels in symptom-free C1-INH-HAE patients were also found to be lower than in healthy controls. Along with others [32] we found this phenomenon in another patient cohort [33]. Demirtürk et al., however, observed this only in type I C1-INH-HAE [32].

In theory danazol treatment could also cause lowering of TNFα levels as it has been found in endometriosis both in vitro and in vivo [16, 34]. This phenomenon has not been observed in C1-INH-HAE.

Unexpectedly, serum fetuin-A levels were increased significantly during the HAE attacks. This finding can be explained by the several observations suggesting fetuin-A plays an inhibitory role in inflammatory processes. Fetuin-A acts as an inhibitor of neutrophil superoxide release [15] and is required for the spermine-induced inhibition of TNFα release of macrophages [35]. Fetuin-A proved to be a specific and potent inhibitor of carrageenan-induced paw edema formation [36]. In accord with this fetuin-A was shown to have a protective role in experimentally induced cerebral ischaemia in rats [37]. This effect was achieved by decrease of local TNFα production, decrease of the infarct size (also associated with brain edema). In addition, high mobility group box 1 (HMGB1), a late-phase proinflammatory cytokine, which is released from ischemic tissues and septic shock increases serum levels of fetuin-A by 2–3-fold [37]. Along with TNFα and IL-1β, HMGB1 also increases vascular permeability [38, 39].

Another explanation for the elevation of fetuin-A levels during the HAE attacks can be linked to the activation of the contact-kinin system, the hallmark of HAE attacks. There are interesting observations on the possible connection between the contact-kinin system and fetuin-A. Bradykinin receptor 1 (BR1) knockout mice have reduced fetuin-A concentration compared to wild type [40]. Furthermore, these mice have lower insulin resistance and are protected from non-alcoholic fatty liver disease (NAFLD) after high fat diet treatment. Fetuin-A is a well-known contributor to the development of insulin resistance and NAFLD [21]. Thus it cannot be ruled out that the activation of the contact-kinin system may result in the upregulation of the fetuin-A synthesis.

These observations suggest that fetuin-A might have a protective role in edema formation of C1-INH-HAE, as well. The increase of serum fetuin-A levels can be explained by its augmented synthesis induced by the damaged endothelium. The biological role of this counter-regulatory action is to protect the endothelial barrier function as it has been demonstrated in animal experiments [36, 37].

In our study we found no significant changes in the CRP levels of C1-INH-HAE patients. This finding is in accord with that of Oshawa, who found normal CRP levels in spite of leukocytosis even during abdominal attacks [41]. Others found elevated CRP levels even in the absence of an attack that increased further mainly in patients with abdominal localization [42]. They suppose that this could be caused by the stimulatory effect caused by translocation of bacterial LPS but the CRP-rising effect of the edema formation itself cannot be ruled out, either [42]. In another series of our patient group (*n* = 26) Veszeli et al. also found that CRP levels were higher during the symptom-free period and, along with true neutrophil activation, increased further during HAE attacks [33]. Different timing of blood sampling can also contribute to the differences in CRP levels in C1-INH-HAE patients. Hofman et al. observed that the rise of CRP occurred early in the attack (i.e., less than 5 h to 1 day) compared to later periods (7 and 22 days) [42]. These findings are in contrast with our result, considering that blood samples were obtained from the patients within 6 h after the onset of the edematous symptoms.

We confirmed that D-dimer levels increased during HAE attacks which have been already described in the literature [43–45].

Case-control design and the relatively small sample size are limitations of our study. Moreover, nine patients were on danazol. Chronic danazol treatment has been found to decrease HDL-cholesterol and increase LDL-cholesterol levels, respectively [46]. This might also be considered as a confounding factor as there is a link between serum fetuin-A levels and blood lipids. Long-term danazol prophylaxis, however, does not deteriorate liver function in patients with HAE [47].

In summary, we found decreased serum fetuin-A concentrations in patients with C1-INH-HAE, which significantly rose during HAE attacks, characteristically in subcutaneous localization. These changes cannot be explained by the negative acute phase character of fetuin-A; rather by the anti-inflammatory characteristics of the protein. Serum levels may not reflect effects of cytokines at the cellular level. Clearly, large scale follow-up studies on different C1-INH-HAE groups are needed to elucidate the behavior and clinical usefulness of fetuin-A, TNFα, and CRP concentrations in this disease.

## Conclusions

Patients with C1-INH-HAE have decreased serum fetuin-A concentrations during the symptom-free period, which is probably not the consequence of the acute phase reaction. Given the anti-inflammatory properties of fetuin-A, the increase of its levels during attacks may contribute to the counter-regulation of edema formation during C1-INH-HAE attacks.

## Abbreviations

C1-INH: C1-inhibitor
C1-INH-HAE: Hereditary angioedema with C1-inhibitor deficiency
CRP: C-reactive protein
LPS: Lipopolysaccharide
NAFLD: Non-alcoholic fatty liver disease
TNFα: Tumor necrosis factor alpha

## References

[1] Nzeako UC, Frigas E, Tremaine WJ (2001). Hereditary angioedema: a broad review for clinicians. Arch Intern Med.

[2] Nussberger J, Cugno M, Cicardi M, Agostoni A (1999). Local bradykinin generation in hereditary angioedema. J Allergy Clin Immunol.

[3] Cugno M, Nussberger J, Cicardi M, Agostoni A (2003). Bradykinin and the pathophysiology of angioedema. Int Immunopharmacol.

[4] Kaplan AP, Joseph K (2010). The bradykinin-forming cascade and its role in hereditary angioedema. Ann Allergy Asthma Immunol.

[5] Bork K, Staubach P, Eckardt AJ, Hardt J (2006). Symptoms, course, and complications of abdominal attacks in hereditary angioedema due to C1 inhibitor deficiency. Am J Gastroenterol.

[6] Zotter Z, Csuka D, Szabo E, Czaller I, Nebenfuhrer Z, Temesszentandrasi G (2014). The influence of trigger factors on hereditary angioedema due to C1-inhibitor deficiency. Orphanet J Rare Dis.

[7] Savarese L, Bova M, De Falco R, Guarino MD, De Luca Picione R, Petraroli A (2018). Emotional processes and stress in children affected by hereditary angioedema with C1-inhibitor deficiency: a multicenter, prospective study. Orphanet J Rare Dis.

[8] Demirtürk M, Polat N, Güz G, Gürdal A, Altun I, Gelincik A (2012). There is an increased risk of atherosclerosis in hereditary angioedema. Int Immunopharmacol.

[9] Firinu D, Bassareo PP, Zedda AM, Barca MP, Crisafulli A, Mercuro G (2018). Impaired endothelial function in hereditary angioedema during the symptom-free period. Front Physiol.

[10] Triffitt JT, Gebauer U, Ashton BA, Owen ME, Reynolds JJ (1976). Origin of plasma alpha-2 HS-glycoprotein and its accumulation in bone. Nature.

[11] Lebreton JP, Joisel F, Raoult JP, Lannuzel B, Rogez JP, Humbert G (1979). Serum concentration of human alpha 2 HS glycoprotein during the inflammatory process: evidence that alpha 2 HS glycoprotein is a negative acute-phase reactant. J Clin Invest.

[12] Jakab L, Jakab L, Kalabay L, Pozsonyi T, Cseh K (1991). The effect of the alpha 2-HS-glycoprotein on the mitogen-induced lymphoblastic transformation and IL-2 production. Acat Physiol Hung.

[13] Lewis JG, Andre CM (1981). Enhancement of human monocyte phagocytic function by alpha 2HS glycoprotein. Immunology.

[14] Jersmann HP, Dransfield I, Hart SP (2003). Fetuin/alpha2-HS glycoprotein enhances phagocytosis of apoptotic cells and macropinocytosis by human macrophages. Clin Sci (Lond).

[15] Terkeltaub RA, Santoro DA. Alpha-2-HS glycoprotein (alpha-2-HSG) is a major regulator of neutrophil (PMN) stimulation by hydroxyapatite (HA) crystals. Clin Res. 1987;35(3):A568-A56A.

[16] Jahnen-Dechent W, Schafer C, Ketteler M, McKee MD (2008). Mineral chaperones: a role for fetuin-a and osteopontin in the inhibition and regression of pathologic calcification. J Mol Med (Berl).

[17] Schafer C, Heiss A, Schwarz A, Westenfeld R, Ketteler M, Floege J (2003). The serum protein alpha(2)-Heremans-Schmid glycoprotein/fetuin-a is a systemically acting inhibitor of ectopic calcification. J Clin Invest.

[18] Dickson IR, Poole AR, Veis A (1975). Localisation of plasma alpha2HS glycoprotein in mineralising human bone. Nature.

[19] Lavebratt C, Wahlqvist S, Nordfors L, Hoffstedt J, Arner P (2005). AHSG gene variant is associated with leanness among Swedish men. Hum Genet.

[20] Reinehr T, Roth CL (2008). Fetuin-a and its relation to metabolic syndrome and fatty liver disease in obese children before and after weight loss. J Clin Endocrinol Metab.

[21] Stefan N, Hennige AM, Staiger H, Machann J, Schick F, Krober SM (2006). Alpha(2)-Heremans-Schmid glycoprotein/fetuin-a is associated with insulin resistance and fat accumulation in the liver in humans. Diabetes Care.

[22] Ix JH, Shlipak MG, Brandenburg VM, Ali S, Ketteler M, Whooley MA (2006). Association between human fetuin-a and the metabolic syndrome: data from the heart and soul study. Circulation.

[23] Dasgupta S, Bhattacharya S, Biswas A, Majumdar SS, Mukhopadhyay S, Ray S (2010). NF-kappaB mediates lipid-induced fetuin-a expression in hepatocytes that impairs adipocyte function effecting insulin resistance. Biochem J.

[24] Ix JH, Wassel CL, Kanaya AM, Vittinghoff E, Johnson KC, Koster A (2008). Fetuin-a and incident diabetes mellitus in older persons. JAMA.

[25] Stefan N, Fritsche A, Weikert C, Boeing H, Joost HG, Haring HU (2008). Plasma fetuin-a levels and the risk of type 2 diabetes. Diabetes.

[26] Laughlin GA, Cummins KM, Wassel CL, Daniels LB, Ix JH (2012). The association of fetuin-a with cardiovascular disease mortality in older community-dwelling adults: the rancho Bernardo study. J Am Coll Cardiol.

[27] Stenvinkel P, Wang K, Qureshi AR, Axelsson J, Pecoits R, Gao P (2005). Low fetuin-a levels are associated with cardiovascular death: impact of variations in the gene encoding fetuin. Kidney Int.

[28] Elzanowski A, Barker WC, Hunt LT, Seibel-Ross E (1988). Cystatin domains in alpha-2-HS-glycoprotein and fetuin. FEBS Lett.

[29] Kellermann J, Haupt H, Auerswald EA, Muller-Ester W (1989). The arrangement of disulfide loops in human alpha 2-HS glycoprotein. Similarity to the disulfide bridge structures of cystatins and kininogens. J Biol Chem.

[30] Yoshioka Y, Gejyo F, Marti T, Rickli EE, Burgi W, Offner GD (1986). The complete amino acid sequence of the A-chain of human plasma alpha 2HS-glycoprotein. J Biol Chem.

[31] Daveau M, Christian D, Julen N, Hiron M, Arnaud P, Lebreton JP (1988). The synthesis of human alpha-2-HS glycoprotein is down-regulated by cytokines in hepatoma HepG2 cells. FEBS Lett.

[32] Demirtürk M, Gelincik A, Cinar S, Kilercik M, Onay-Ucar E, Colakoglu B (2014). Increased eNOS levels in hereditary angioedema. Int Immunopharmacol.

[33] Veszeli N, Csuka D, Zotter Z, Imreh E, Jozsi M, Benedek S (2015). Neutrophil activation during attacks in patients with hereditary angioedema due to C1-inhibitor deficiency. Orphanet J Rare Dis.

[34] Liu Y, Luo L, Zhao H (2000). In vitro effect of danazol on cytokine production of macrophages in peritoneal fluid of infertile patients with endometriosis and its relationship with cytosolic free calicum concentration. Zhonghua Fu Chan Ke Za Zhi.

[35] Wang H, Zhang M, Soda K, Sama A, Tracey KJ (1997). Fetuin protects the fetus from TNF. Lancet.

[36] Ombrellino M, Wang H, Yang H, Zhang M, Vishnubhakat J, Frazier A (2001). Fetuin, a negative acute phase protein, attenuates TNF synthesis and the innate inflammatory response to carrageenan. Shock.

[37] Wang H, Li W, Zhu S, Li J, D'Amore J, Ward MF (2010). Peripheral administration of fetuin-a attenuates early cerebral ischemic injury in rats. J Cereb Blood Flow Metab.

[38] Ong SP, Lee LM, Leong YF, Ng ML, Chu JJ (2012). Dengue virus infection mediates HMGB1 release from monocytes involving PCAF acetylase complex and induces vascular leakage in endothelial cells. PLoS One.

[39] Wakamoto S, Fujihara M, Sakagawa H, Takahashi D, Niwa K, Morioka M (2008). Endothelial permeability is increased by the supernatant of peripheral blood mononuclear cells stimulated with HLA class II antibody. Transfusion.

[40] Fonseca RG, Sales VM, Ropelle E, Barros CC, Oyama L, Ihara SS (2013). Lack of kinin B(1) receptor potentiates leptin action in the liver. J Mol Med (Berl).

[41] Ohsawa I, Nagamachi S, Suzuki H, Honda D, Sato N, Ohi H (2013). Leukocytosis and high hematocrit levels during abdominal attacks of hereditary angioedema. BMC Gastroenterol.

[42] Hofman ZL, Relan A, Hack CE (2014). C-reactive protein levels in hereditary angioedema. Clin Exp Immunol.

[43] van Geffen M, Cugno M, Lap P, Loof A, Cicardi M, van Heerde W (2012). Alterations of coagulation and fibrinolysis in patients with angioedema due to C1-inhibitor deficiency. Clin Exp Immunol.

[44] Csuka D, Veszeli N, Imreh E, Zotter Z, Skopal J, Prohaszka Z (2015). Comprehensive study into the activation of the plasma enzyme systems during attacks of hereditary angioedema due to C1-inhibitor deficiency. Orphanet J Rare Dis..

[45] Reshef A, Zanichelli A, Longhurst H, Relan A, Hack CE (2015). Elevated D-dimers in attacks of hereditary angioedema are not associated with increased thrombotic risk. Allergy.

[46] Szeplaki G, Varga L, Valentin S, Kleiber M, Karadi I, Romics L (2005). Adverse effects of danazol prophylaxis on the lipid profiles of patients with hereditary angioedema. J Allergy Clin Immunol.

[47] Farkas H, Czaller I, Csuka D, Vas A, Valentin S, Varga L (2010). The effect of long-term danazol prophylaxis on liver function in hereditary angioedema-a longitudinal study. Eur J Clin Pharmacol.

